# Interactions between temperature and drought in global and regional crop yield variability during 1961-2014

**DOI:** 10.1371/journal.pone.0178339

**Published:** 2017-05-26

**Authors:** Michael Matiu, Donna P. Ankerst, Annette Menzel

**Affiliations:** 1 Ecoclimatology, Technical University of Munich, Freising, Germany; 2 Department of Mathematics, Technical University of Munich, Garching, Germany; 3 Institute for Advanced Study, Technical University of Munich, Garching, Germany; Instituto Agricultura Sostenible, SPAIN

## Abstract

Inter-annual crop yield variation is driven in large parts by climate variability, wherein the climate components of temperature and precipitation often play the biggest role. Nonlinear effects of temperature on yield as well as interactions among the climate variables have to be considered. Links between climate and crop yield variability have been previously studied, both globally and at regional scales, but typically with additive models with no interactions, or when interactions were included, with implications not fully explained. In this study yearly country level yields of maize, rice, soybeans, and wheat of the top producing countries were combined with growing season temperature and SPEI (standardized precipitation evapotranspiration index) to determine interaction and intensification effects of climate variability on crop yield variability during 1961–2014. For maize, soybeans, and wheat, heat and dryness significantly reduced yields globally, while global effects for rice were not significant. But because of interactions, heat was more damaging in dry than in normal conditions for maize and wheat, and temperature effects were not significant in wet conditions for maize, soybeans, and wheat. Country yield responses to climate variability naturally differed between the top producing countries, but an accurate description of interaction effects at the country scale required sub-national data (shown only for the USA). Climate intensification, that is consecutive dry or warm years, reduced yields additionally in some cases, however, this might be linked to spillover effects of multiple growing seasons. Consequently, the effect of temperature on yields might be underestimated in dry conditions: While there were no significant global effects of temperature for maize and soybeans yields for average SPEI, the combined effects of high temperatures and drought significantly decreased yields of maize, soybeans, and wheat by 11.6, 12.4, and 9.2%, respectively.

## Introduction

Climate change alters global food production, with impacts dependent upon crop, region, magnitude of warming, changes in precipitation patterns and extreme events, production technology, and adaptation measures [1]. Past evidence has shown climate change to more likely incur damage rather than draw benefits for crop yields [2–5], with induced yield losses only able to be partly offset by endogenous economic responses [6].

The intention to mitigate climate change took a significant step forward during the 2015 United Nations Climate Change Conference in Paris. But irrespective of the future success of such efforts, year-to-year climate variability is unlikely to diminish, and hence neither will its impacts on food [7]. Climate variability accounts for up to 60% of yield variability in major parts of the world [8] and is thus an important factor in food stability. Low yield variability is desirable, because it leads to more stable food production and farmer income [7]. However, changes in yield variability have been positively associated with changes in climate variability [9], suggesting that food stability might continue to deteriorate if climate variability continues to increase, for example, as a consequence of failures to mitigate climate change [10].

The most influential climate variables affecting yields on a global scale are temperature and precipitation, with some studies indicating that they act nonlinearly [2,8,11–15]. Interactions between temperature and precipitation might lead to reduced sensitivity to heat if enough water is available [16], and thus higher relative importance of heat [17]. So while the importance of climate interactions is acknowledged [12] and in some cases included in the models [8,17,18], they are rarely described in detail because of their complexity. This study proposes a way to visualize interaction effects, and quantify interacting effects by conditioning one variable on quantiles of the other. Another type of climate interactions are intensification effects from consecutive dry or warm years [19], which can be assessed by interaction terms of climate variables with their lagged values from previous years.

To this end, this study aims to quantify the interaction between temperature and drought variability in crop yield variability for the four most important crops worldwide (maize, rice, soybeans, and wheat) both at the global and country scale, in order to assess the (1) effects of temperature and drought interactions on yield, (2) differences between crops, and (3) differences between the global climate sensitivities and that for major producers.

## Materials and methods

Yearly country-level data on crop production (tons from 1961 to 2014) and harvested area (ha from 1961 to 2014) was available from the Food and Agriculture Organization of the United Nations (FAO, available at http://faostat.fao.org/default.aspx). Yield was then defined as the ratio of production and harvested area. Additionally, the FAO provided globally aggregated data on world production of crops (in tons from 1961–2014, FAO), which was used to determine the top producing countries. The focus was on the primary staple crops that constitute large parts of the human diet: maize, rice, soybeans and wheat, and restricted to countries that had at least an average share of 1% to global production during 1961–2014.

FAO data is annually reported separately by each country, with the consequence that data quality might be inhomogeneous. If countries reported exactly the same values of production for two or more consecutive years, only data from the years after or before the last occurrence of identical values were used (Argentina maize until 2011, India soybeans from 1972 onwards, United Kingdom wheat from 1991 onwards). Furthermore, yield time series that showed constant trends over multiple years, were also discarded (Iran wheat, Turkey wheat) and some extremely improbable values were removed (~1/10 production of France maize in 2014 compared to 2013; double or half yields from one year to the next of Paraguay soybeans before 1969). A summary of the FAO data used for the analysis can be found in S1 Table.

Monthly temperature on a 0.5° grid was taken from the CRU TS 3.23 data set [20], while 1-month SPEI (Standardized Precipitation Evapotranspiration Index) values on the same temporal and spatial resolution were obtained from the SPEIbase v.2.4 [21], which is also based on the CRU TS 3.23 data set. The SPEI is calculated by taking the difference between precipitation and potential evapotranspiration and thus including the impact of temperature on water demand. Values are then standardized for each month and location using log-logistic distributions. Using precipitation instead of SPEI produced qualitatively similar results but lower accuracy. Explanations for the latter could be that the SPEI describes wet- and dryness more accurately on a global scale since it accounts for the varying rates of evapotranspiration as well as being standardized. Including both in the modelling induced collinearity, since precipitation and SPEI were highly correlated, thus only SPEI was used.

In order to merge the climate and crop data, the climatic variables were aggregated to match the temporal (yearly) and spatial (country) resolution of the crop yields in a two-step procedure. First, the day-of-year of planting and harvesting from the crop calendar of Sacks *et al*. [22] was used to derive yearly growing season means of temperature and SPEI for each 0.5° grid. Averages were calculated using all monthly climate values between the days of planting and harvesting; for example, if planting was March 2 (or 29) and harvesting September 23 (or 5), monthly temperatures and SPEI from March to September inclusive were included in the average. Second, the 0.5° grid growing season averages were aggregated to crop-area weighted country means, for which crop area weights were taken from planted area estimates [23]. While there is some evidence of advancing planting dates in the recent decades, for example in the central USA maize is planted two weeks earlier compared to when it was routinely planted in the early 1980s [24], for other regions like central Europe advancements in crop planting dates are less prominent (e.g. only 0.4 days earlier per decade) [25], consequently using monthly climate data adjusted to a fixed cropping season still seemed appropriate.

Maize, rice and wheat are all grown in multiple seasons. For maize only the main season was used, since the second season constituted a non-significant share of total yields. For rice, the second season contributed large shares to total yields in some countries, so yields were averaged over the two growing season climates, with weights as given in [26]. Since the distinction of winter and spring wheat in the crop calendar was somewhat arbitrary, and as wheat is dormant and rather insensitive to climate conditions in winter, the four months before harvest of the main season were used as the growing season [2].

The logarithm was applied to yields, which turns absolute into relative effects, since climate affects yield in relative and not absolute terms. In other words a 1°C difference in temperature should have the same effect irrespective if yields are 5 ton/ha or 1 ton/ha. Using logged yield is standard practice [2,4,13–15], and also removes the issues of the skewed yield distribution and heteroscedascity (increased yield variance for higher yields).

Since the focus is on climate variability and its effects on yield variability, trends in climate and yield could confound the estimated relationship and induce spurious correlations if concurrent trends existed. Thus yield, temperature and SPEI were detrended using separate models for each crop-country combination. For temperature and SPEI, penalized regression splines (mgcv-package in R) were used, with a maximum basis dimension of 5 (the actual basis dimension is determined by cross-validation) and the possibility to penalize to zero when there is no trend. The length of the yield time-series varied between 23 and 54, so the maximum basis dimension was set to number of years divided by ten, but not below 3. Inspecting residuals, some crop-country yield time series (S2 Table) were poorly fit, so in order to have appropriate models the basis dimension was doubled. This flexible approach was chosen over linear, quadratic or cubic trends, because it could handle multiple types of non-linearity and removed the need for selecting the most appropriate polynomial.

For each crop, detrended time-series data of all countries were included into one mixed model to explain log detrended yields:

$$\log\;dYield_{c,t}=(\alpha +\alpha_{c})+(\beta +\beta_{c})Climate_{c,t}+(\gamma +\gamma_{c})Climate.Interactions_{c,t}+(\delta +\delta_{c})Climate.Intensification_{c,t,t-1}+\epsilon_{c,t}$$

where

- *c* is a country index and *t* is for year (1961–2014).
- *dYield*_*c*,*t*_ is the detrended yield in country *c* and year *t*.
- *Climate*_*c*,*t*_ consists of detrended temperature (*dTemp*) and SPEI (*dSPEI*), as well as quadratic terms which implied optimal temperatures/SPEI for yield, while permitting negative effects for low and/or high temperatures or SPEI values: $(\beta +\beta_{c})Climate_{c,t}=(\beta_{1}+\beta_{1,c})dTemp_{c,t}+(\beta_{2}+\beta_{2,c})dTemp_{c,t}^{2}+(\beta_{3}+\beta_{3,c})dSPEI_{c,t}+(\beta_{4}+\beta_{4,c})dSPEI_{c,t}^{2}$.
- *Climate*.*Interactions*_*c*,*t*_ are interaction terms between detrended temperature and SPEI. The interaction terms accommodated different temperature effects depending on SPEI, for example, allowing a 1°C change in temperature to have a different impact on yield for dry compared to wet conditions: $(\gamma +\gamma_{c})Climate.Interactions_{c,t}=(\gamma_{1}+\gamma_{1,c})dTemp_{c,t}dSPEI_{c,t}+(\gamma_{2}+\gamma_{2,c})dTemp_{c,t}^{2}dSPEI_{c,t}+(\gamma_{3}+\gamma_{3,c})dTemp_{c,t}dSPEI_{c,t}^{2}+(\gamma_{4}+\gamma_{4,c})dTemp_{c,t}^{2}dSPEI_{c,t}^{2}$.
- *Climate*.*Intensification*_*c*,*t*,*t-1*_ are previous year temperature and SPEI, and their interaction terms with current year values. These allow for intensification effects of consecutive warm, cold, dry, or wet years: (*δ* + *δ*_*c*_)*Climate.Intensification*_*c,t,t*−1_ = (*δ*_1_ + *δ*_1,*c*_)*dTemp*_*c,t*−1_ + (*δ*_2_ + *δ*_2,*c*_)*dSPEI*_*c,t*−1_ + (*δ*_3_ + *δ*_3,*c*_)*dTemp*_*c,t*−1_*dTemp*_*c,t*_ + (*δ*_4_ + *δ*_4,*c*_)*dSPEI*_*c,t*−1_*dSPEI*_*c,t*_.

In the above model, α is the global intercept, and β, γ, and δ are slopes for climate variables, while coefficient vectors with subscript *c* accommodate different sensitivities for each country using a random effects specification, that is (*α*_*c*_,*β*_*c*_,*γ*_*c*_,*δ*_*c*_)∼*N*(0,Σ) with $\Sigma =I_{13}(\sigma_{1}^{2},\ldots ,\sigma_{13}^{2})$ and I_13_ identity matrix of dimension 13. Residual variability was not homogenous across countries, thus a different error variance per country was included, that is *Var*(*ϵ*_*c,t*_) = *σ*^2^*ϕ*_*c*_ with estimated variance ratios *ϕ*_*c*_(*c* ≥ 2) relative to the first country with *ϕ*_1_ = 1. To arrive at a parsimonious description, non-significant variables were removed.

To compare global climate sensitivities to country effects, single country time series were modelled using the same variables as above (without random effects) for the five top producers of each crop (Table 1). For model selection, non-significant variables were excluded until the minimum BIC (Bayesian Information Criterion) was attained.

**Table 1:** The top five producers by crop as of 2014.

Crop	Country	Production [million ton]	Share in global production [%]
Maize	USA	361	29.2
	China	216	17.4
	Brazil	80	6.5
	Ukraine	28	2.3
	India	24	1.9
Rice	China	208	21.9
	India	157	16.6
	Indonesia	71	7.5
	Bangladesh	52	5.5
	Viet Nam	45	4.7
Soybeans	USA	108	33.7
	Brazil	87	27.1
	Argentina	53	16.7
	China	12	3.8
	India	11	3.3
Wheat	China	126	14.8
	India	94	11.0
	Russia	60	7.0
	USA	55	6.5
	France	39	4.6

For each crop, interacting climate effects were evaluated as fitted values holding all other variables constant that were not part of the interaction. For example, to show the effects of temperature and SPEI, the fitted values for temperature were evaluated at three quantiles of the SPEI distribution denoting extreme dry (0.05 quantile), normal (0.50, median), and extreme wet (0.95) conditions. Similarly climate intensification effects were evaluated over current year temperature/SPEI given three quantiles (as above) of previous year temperature/SPEI. The effects (fitted values) on the log scale were exponentiated, so they became ratios, and then one was subtracted so they became relative differences.

Robustness of the models was evaluated by cross validation, specifically by LOOCV (leave-one-out-cross-validation). Additionally LOOCV errors were calculated for models without interaction terms (but where variables could be linear or quadratic), and for models where all variables were included only linearly (thus without interactions).

As a sensitivity analysis, state-level data for the USA was used to derive the national sensitivity of yields to climate using the same random effects specification as above for the global sensitivity. State-level yields (bu/acre) for maize, soybeans, and wheat were available from the National Agricultural Statistics Service of the United States Department of Agriculture (Quick Stats, available at: https://www.nass.usda.gov/Quick_Stats/index.php) for the same study period (1961–2014). No data had to be removed using the quality criteria adopted above for the FAO data. To ensure comparability, the same work-flow procedure was adopted: state-level climate was derived using the same data sources (the crop calendar data contains information at the state scale); detrending and modelling as above; although yield units differ (bu/acre vs t/ha), modelling results are on the %-scale, so no conversion was needed. For the yield detrending of the state time series, three crop-state combinations had to have double the basis dimension (Arizona maize, Maryland wheat, and Oregon maize).

State level yields and climate variables of the USA were aggregated to country averages using the production in each state as weights. Then the same models as for country level data from the FAO were run, in order to compare results obtained from sub-country data to results from country averages.

To quantitatively assess the potential impact of measurement error in FAO yield data on statistical significance of higher-order effects in the model, such as interactions, a simulation study was performed. Noise was added to the detrended log yields by calculating the standard deviation (sd) of each crop-country time series and then adding normally distributed random noise with mean 0 and sd ranging from 1, 2, 3 … to 50% of the initial sd to ensure the same percent relative error across the different crop by country time series. The original models were then refit to the noisy data. The addition of random noise was repeated 100 times for each %-level of added noise, yielding 100 simulations of p-values corresponding to the significance of the highest order term(s), whether they being interaction, quadratic, or linear terms, depending on model. The 100 replications of p-values corresponding to a specific percent relative noise were presented in terms of stacked bar charts.

## Results

What follows is a description of the interaction effects found globally and for the five top producing countries, discussed in turn for each crop, followed by an assessment of the intensification effects by previous year climate variability. Then a sensitivity analysis of using state-level data is presented for the USA.

If not otherwise stated, effects for high and low temperature and SPEI are for the respective 5 and 5% quantiles. Percent effects on yields are followed by 95% confidence intervals in brackets, or ns if not significant.

### Effects of climate variability and interactions on crop yields

#### Maize

**Fig 1:**
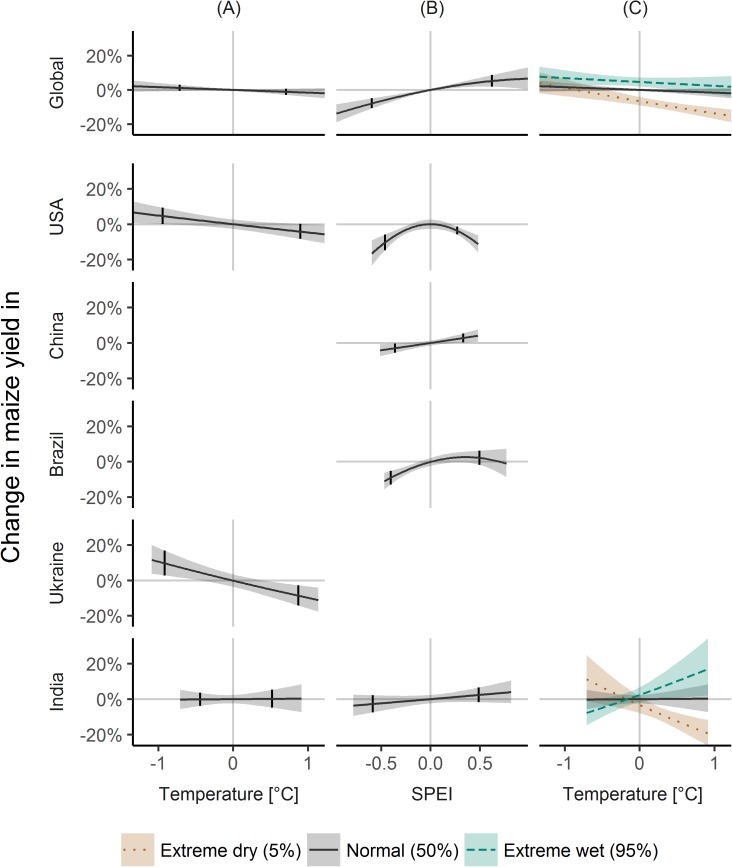
Climate variability effects on maize yield variability. Black lines show the effects of changes in detrended temperature (A, C) and changes in SPEI (standardized precipitation evapotranspiration index, B) on changes in mean maize yield globally and for the five top producing countries. Increasing lines mean that higher temperatures (A, C) or SPEI (B) were associated with greater increases in mean yields, while decreasing lines imply association with decreases in mean yields. Dashed and dotted lines in (C) indicate significant interaction effects. All lines are estimates from the regression models, and the absence of a line indicates non-significance of an association. Shades around the lines indicate pointwise confidence intervals for the mean change in maize yield as estimated from the regression models. Small vertical lines denote the 5 and 95% quantile of detrended temperature (A) and SPEI (B). Countries are ordered according to their total production from the top producer USA downwards.

Globally, maize yields decreased by -7.8% (-10.7, -4.9) in dry and increased by 5.2% (1.9, 8.7) in wet conditions for average temperatures (Fig 1B), but temperature was non-significant for average SPEI (Fig 1A). However, considering interactions, higher temperatures were linked to decreased yields under dry conditions of -11.6% (-14.3, -8.9), but not under wet conditions (Fig 1C).

For the USA, the top producer of maize, low and high temperatures were linked to yield changes of 4.7% (0.1, 9.5) and -4.1% (-8.3, 0.2) (Fig 1A), and both dry and wet conditions were associated to yield decreases, however, stronger for dry with -10.4%, (-14.8, -5.9) than wet with -3.5%, (-5.7, -1.3) (Fig 1B).

For China, no significant temperature effect was found, only a modest effect of -3.0% (-5.5, -0.4) of dry and 2.8% (0.2, 5.4) of wet conditions (Fig 1B).

For Brazil, only dry conditions were associated to yield reductions of -9.1% (-12.9, -5.2), while wet conditions and temperature were not significant (Fig 1A and 1B).

For Ukraine, temperature variability was negatively associated to maize yield variability with 9.6% (2.8, 16.8) for cold and -8.6% (-14.1, -2.7) for warm conditions (Fig 1A).

For India, the temperature-SPEI interaction was highly significant. While SPEI had no significant effect at average temperatures (Fig 1A), for high temperatures dry conditions were associated to yield decreases of -12.7% (-17.2, -8.0) and wet conditions to yield increases of 10.6% (0.9, 21.2) (Fig 1C).

#### Rice

**Fig 2:**
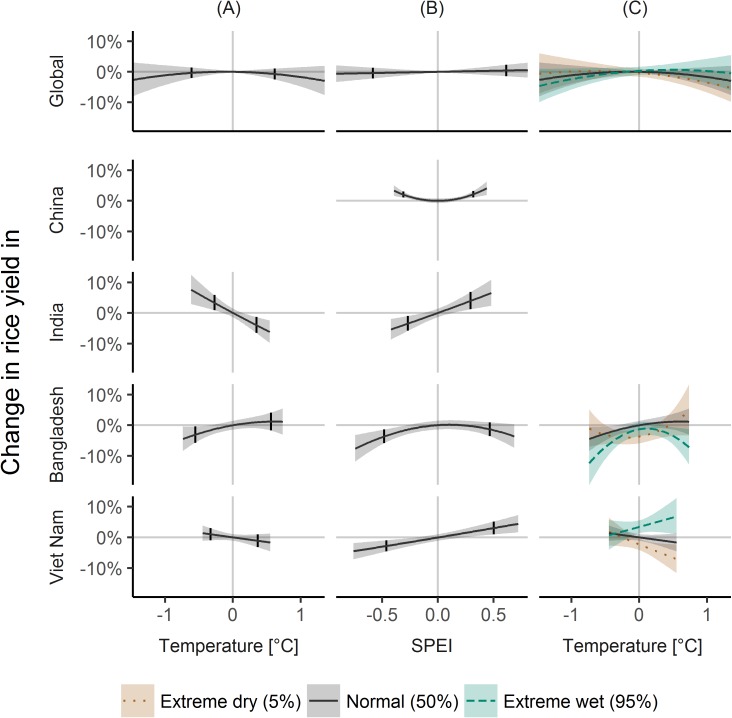
Climate variability effects on rice yield variability. Same as Fig 1, but for rice. Indonesia omitted, because of no significant effects to show.

Globally, rice yield variability showed some dependence on temperature and SPEI variability (Fig 2A–2C), however, effects between the 5 and 95% quantile of climate variables were non-significant at p = 0.05. However, at the country scale, effects of climate variability were clearer.

For China, temperature variability was not significant (Fig 2A), but dry and wet conditions were associated to yield increases of 2.0% (1.0, 3.0) and 2.2% (1.1, 3.2), respectively (Fig 2B).

For India, high temperatures and dry conditions were associated to yield decreases of -3.9% (-6.5, -1.3; Fig 2A) and -3.4% (-5.8, -1.0; Fig 2B). On the other hand, low temperatures and wet conditions were associated to yields increases of 3.4% (0.9, 5.9; Fig 2A) and 4.0% (1.2, 6.9; Fig 2B).

For Bangladesh, low temperatures and dry conditions were linked to yield decreases of -3.1% (-5.8, -0.4; Fig 2A) and -3.7% (-5.9, -1.4; Fig 2B) for average SPEI and temperature conditions, respectively. Considering interacting effects, extreme wet conditions were linked to yield decreases for both low and high temperatures of -8.3% (-13.0, -3.5) and -4.3% (-7.6, -0.8) but not for average temperatures (Fig 2C).

For Viet Nam, dry and wet conditions were linked to yield changes of -2.8% (-4.6, -0.9) and 3.0% (1.0, 5.2) (Fig 2B), while temperature was non-significant for average SPEI (Fig 2A). However, because of interactions, high temperatures were associated to yield changes of -5.5% (-8.7, -2.1) for dry conditions and 5.6% (1.6, 9.9) for wet conditions (Fig 2C).

#### Soybeans

**Fig 3:**
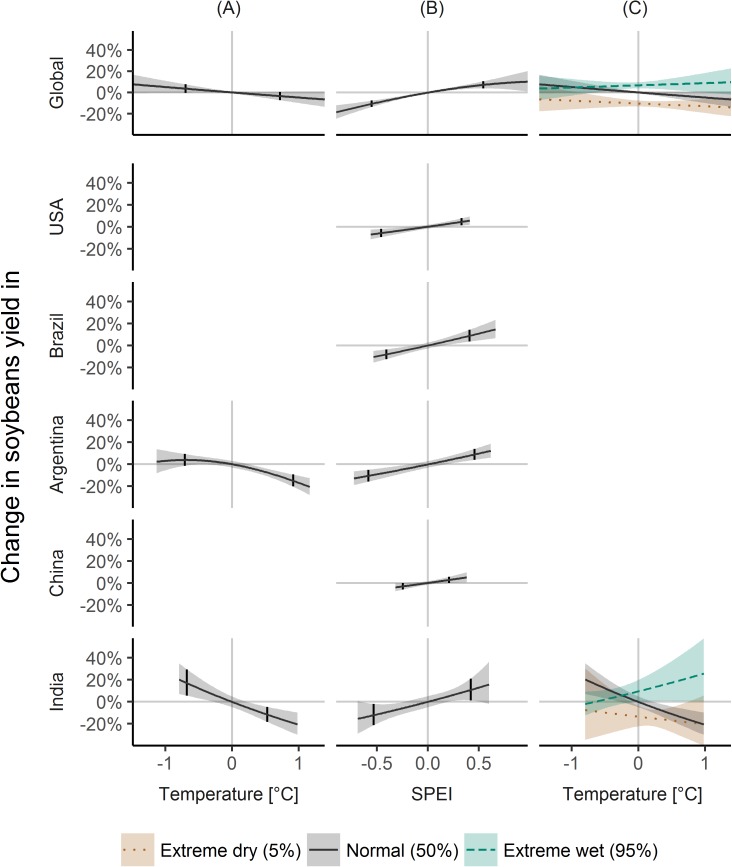
Climate variability effects on soybeans yield variability. Same as Fig 1, but for soybeans.

Globally, soybeans yield variability was more associated to SPEI variability, with yield effects of 7.1% (3.8, 10.6) and -10.7% (-13.6, -7.7) for wet and dry conditions, respectively (Fig 3B). The effect of temperature was small (Fig 3A), as well as the interaction effect, leading for example to yield decreases of -12.4% (-17.1, -7.4) for hot and dry conditions (Fig 3C).

For the USA, only SPEI was significant, and yields effects were -5.7% (-9.4, -2.0) for dry conditions and 4.5% (1.2, 7.8) for wet conditions (Fig 3B).

For Brazil, soybean yields were also only affected by dry and wet conditions with -8.1% (-12.5, -3.5) and 8.8% (3.3, 14.4), respectively (Fig 3B).

For Argentina, high temperatures were associated to yield decreases of -14.9% (-20.2, -9.3), while the effect of low temperatures was non-significant (Fig 3A). Dry and wet conditions were linked to -10.7% (-15.9, -5.0) and 8.8% (4.0, 13.9) yield changes (Fig 3B).

For China, only SPEI was significantly linked to soybean yields, with -3.1% (-5.9, -0.1) and 2.8% (0.0, 5.7) changes in yields for dry and wet conditions (Fig 3B).

For India, temperature variability was negatively associated to soybean yields with 16.8% (5.4, 29.3) for cold and -11.8% (-18.4, -4.7) for warm conditions (Fig 3A), and SPEI was positively associated with -12.2% (-21.4, -2.0) for dry and 10.7% (1.2, 21.0) for wet conditions (Fig 3B). However, the combined influence resulted in yield effects of -17.3% (-28.5, -4.4) for hot and dry, and 18.0% (0.6, 38.3) for hot and wet, and non-significant effects for cold and dry, and cold and wet (Fig 3C).

#### Wheat

**Fig 4:**
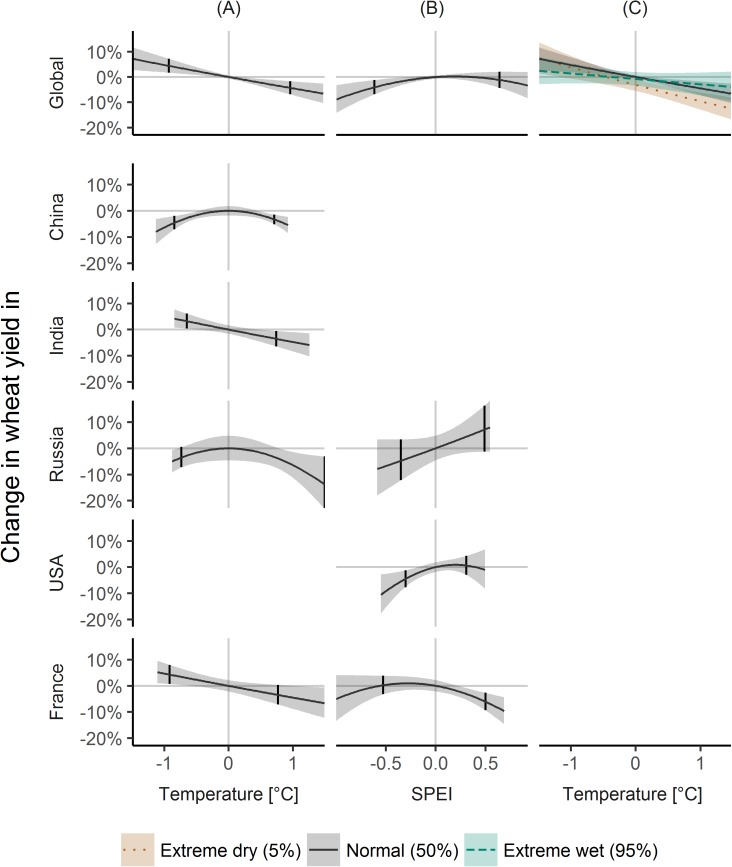
Climate variability effects on wheat yield variability. Same as Fig 1, but for wheat.

Globally, wheat yields were changed by 4.4% (1.7, 7.2) for cold, by -4.2% (-6.8, -1.6) for warm (Fig 4A), by -4.0% (-6.8, -1.1) for dry, and non-significant for wet conditions (Fig 4B). Interaction effects lead to increased effects of -9.2% (-12.4, -5.9) of high temperature under dry conditions and non-significant temperature effects under wet conditions (Fig 4C).

For China, both low and high temperatures were linked to yield decreases of -4.5% (-7.0, -1.9) and -3.3% (-5.1, -1.4) (Fig 4A).

For India, low and high temperatures were linked to yield changes of 3.2% (0.4, 6.2) and -3.5% (-6.5, -0.5) (Fig 4A).

For Russia, high temperatures were linked to yield decreases of -13.5% (-22.9, -3.1; Fig 4A), while low temperatures and SPEI were non-significant at p = 0.05 (Fig 4A and 4B).

For the USA, dry conditions were associated to yield decreases of -4.5% (-7.7, -1.3), and above average SPEI was non-significant (Fig 4B).

For France, cold temperatures were associated to yield increases of 4.3% (0.7, 7.9; Fig 4A), and wet conditions to yield decreases of -6.0% (-9.3, -2.6; Fig 4B).

### Effects of previous year climate variability

**Fig 5:**
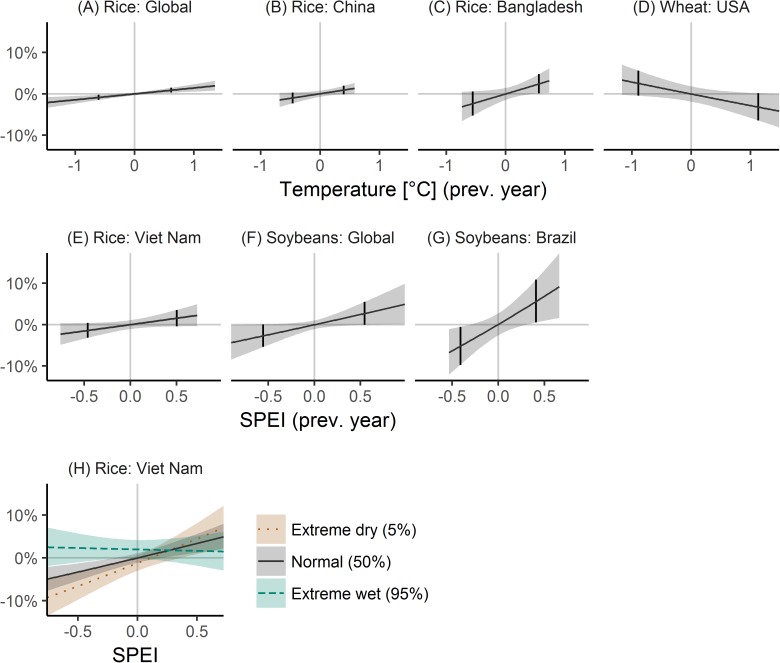
Previous year climate variability effects on crop yield variability. The figure shows how detrended crop yields are affected by previous year (lag) detrended temperature (A-D) and previous year SPEI (standardized precipitation evapotranspiration index, E-G). For rice in Viet Nam also interaction effects with current year climate variability are shown (that is yield effects of current year detrended SPEI given the 5% and 95% quantile of previous year detrended SPEI, H). Small vertical lines denote the 5 and 95% quantile of detrended temperature and SPEI. Crop-country combinations are missing if previous year temperature and SPEI were non-significant. Shaded areas correspond to 95% confidence intervals.

Previous year climate variability was associated globally to rice and soybeans yield variability, and for selected countries to rice, soybeans and wheat yield variability (Fig 5). For rice, previous year temperature was positively associated to yields, such that warm temperatures increased next year yields by 0.9% (0.3, 1.5) globally (Fig 5A), by 0.9% (-0.1, 2.0) for China (Fig 5B), and by 2.4% (0.1, 4.7) for Bangladesh (Fig 5C). Rice yields in Viet Nam were positively associated to previous year SPEI (Fig 5E), and interactions between the previous and current year SPEI resulted in additionally decreased yields for dry conditions if the previous year was also dry with total yield effects of -6.2% (-9.2, -3.1), and no significant effect of current year SPEI, if the previous year was wet (Fig 5H). Soybean yields were linked positively to previous year SPEI with wet conditions followed by yield increases of 2.7% (-0.1, 5.5) globally (Fig 5F) and of 5.6% (0.5, 10.9) for Brazil (Fig 5G). Wheat yields in the USA were negatively associated to previous year temperature, and high temperatures reduced yields in the following year by -3.2% (-6.5, 0.1) (Fig 5D).

Overall, far fewer climate interactions were significant for single country time series than for the global sensitivity. To check, whether this might be related to the low number of observations available to determine single country sensitivities as opposed to pooling the countries using a random effects specification to determine global sensitivities, the analysis was repeated for the USA, but using state-level data.

### Sensitivity check USA: Interactions determined from state level yields

**Fig 6:**
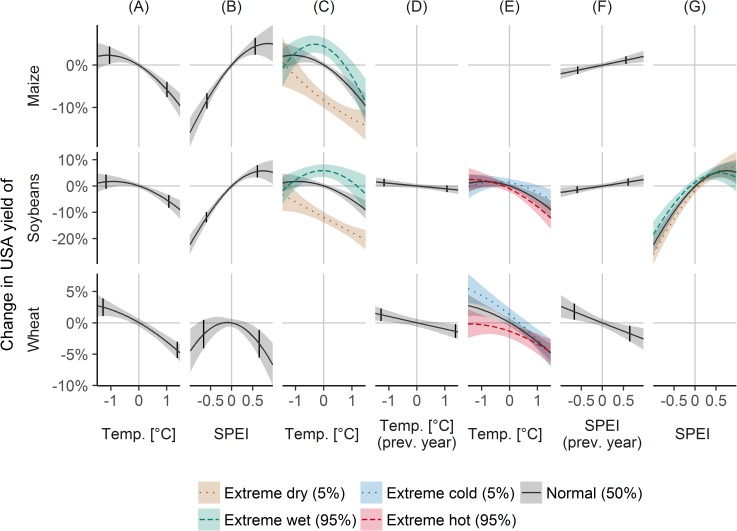
Climate variability effects on crop yield variability for the USA, determined by state level yield statistics. The figure shows how detrended USA yields are affected by detrended temperature (A), SPEI (standardized precipitation evapotranspiration index, B), previous year temperature (D) and previous year SPEI (F) given mean conditions; and how the temperature sensitivity differs by SPEI (for extreme dry and wet conditions denoted by the 5% and 95% quantile of SPEI, C), how the temperature sensitivity differs by previous year temperature (for extreme cold and hot conditions denoted by the 5% and 95% quantiles, E), and how the SPEI sensitivity differs by previous year SPEI (G). Black lines indicate main effects from the regression models, dashed and dotted lines indicate interaction effects, and absence of lines mean absence of significant effects. Small vertical lines denote the 5 and 95% quantile of detrended temperature and SPEI. Shaded areas correspond to 95% confidence intervals.

As a comparison, instead of using country averages for yields and climate, for the USA, data at the state level were used to estimate the national yield sensitivity to climate variability (Fig 6). Effects of climate variability were naturally more detailed, but also more interactions were observed than with only country averages.

For maize, dry conditions and high temperatures reduced yields by -8.5% (-10.3, -6.6; Fig 6B) and -5.8% (-7.6, -3.9; Fig 6A), but lower than average temperature and higher than average SPEI only had small effects on yields that slowly levelled off (Fig 6A and 6B). Regarding interactions, dry conditions reduced yields less at high and low temperatures than at average temperatures, however, yield losses for dry and warm conditions amounted to -12.6% (-14.9, -10.2) (Fig 6C). Wet conditions had positive effects of 4.4% (2.4, 6.4) at average temperatures (Fig 6B), but non-significant effects at low and high temperatures (Fig 6C).

For soybeans, dry conditions and high temperatures were associated to yield decreases of -11.9% (-13.9, -9.9; Fig 6B) and -5.9% (-8.3, -3.4; Fig 6A) for average conditions, and their combined effect amounted to -18.2% (-20.8, -15.5; Fig 6C). Similar to maize, wet conditions were linked to yield increases of 5.5% (3.2, 7.9) only for average temperatures, but not for low and high temperatures (Fig 6C). Previous year temperature had negative effects on yields (Fig 6D) and SPEI positive (Fig 6F), but because of interactions, these effects were only present if the current year was warm or dry, but not if it was cold or wet (Fig 6E and 6G).

For wheat, yield decreases associated with high temperatures were -4.3% (-5.6, -3.0; Fig 6A), with dry conditions -1.8% (-4.1, 0.5) and with wet conditions -3.4% (-5.6, -1.1) (Fig 6B), and yield increases associated with low temperatures were 2.5% (1.1, 3.9; Fig 6A). Previous year temperature and SPEI were negatively associated with wheat yields (Fig 6D and 6F). Because of interactions of current and previous year temperature, the negative effect of previous year temperatures held only if the current year was cold, but not if warm (Fig 6E).

Next, climate variability effects determined by state level data were compared to effects estimated from country averages, which were aggregated from state-level yields and climate (S1 Fig). While they agreed in general, estimating country effects from state-level data was more useful, in that it contained more detailed climate effects. For maize, yield reductions from dry and warm conditions were of similar magnitude if determined from national or state level data, however, the national level data showed negative effects of wet conditions, which were not present for state level data. For soybeans, yield effects of dry and wet conditions agreed between national and state level data, but with national data, effects of temperature, interactions of temperature and SPEI, and previous year temperature were lacking. For wheat, national and state level data agreed for the effect of dry conditions and temperature lag, but disagreed for the effect of wet conditions. Also state level data showed additional effects of temperature, interaction of current and previous year temperatures, and the previous year SPEI.

### Model validation and simulation of errors in FAO yield data

To check the accuracy and robustness of the presented model results, cross validation errors were computed for a suite of models (Table 2), that include the original models and models without interactions, as well as models where all terms are included only linearly. For the global models, RMSE (root mean square errors) of the full models were lower than that of the simpler models for maize and wheat, while for rice and soybeans, RMSEs were similar. For the country level models, removing interactions and/or quadratic effects resulted in larger errors across all crops and countries. Using state-level yields for the USA models resulted in similar errors for the full and no interaction models, and higher errors for linear only models.

**Table 2:** Cross validation results. Reported are root mean square error (RMSE) and median absolute error (MAE) of leave-one-out-cross-validation (LOOCV) of each model presented in the study (Full), of models without interaction terms (NoInter), and where additionally all variables enter only linearly (OnlyLin). OnlyLin models are thus nested within NoInter, which are nested within Full. If nested models had the same formula as the more complex model (e.g. no interactions in the full model or only linear terms when interactions were removed), cells were left empty.

Crop	Level	RMSE			MAE		
		Full	NoInter	OnlyLin	Full	NoInter	OnlyLin
Maize	Global	0.118	0.119	0.120	0.048	0.050	0.050
Rice	Global	0.045	0.045	0.045	0.023	0.023	0.024
Soybeans	Global	0.113	0.113	0.113	0.063	0.060	0.063
Wheat	Global	0.108	0.108	0.111	0.051	0.052	0.052
Maize	Brazil	0.069		0.072	0.040		0.049
	China	0.052			0.040		
	India	0.082	0.089		0.058	0.064	
	USA	0.087		0.095	0.059		0.068
	Ukraine	0.086			0.057		
Rice	Bangladesh	0.037	0.039	0.041	0.023	0.021	0.027
	China	0.022		0.023	0.016		0.015
	India	0.053			0.035		
	Viet Nam	0.042	0.046		0.025	0.023	
Soybeans	Argentina	0.094		0.097	0.052		0.051
	Brazil	0.100			0.066		
	China	0.061			0.034		
	India	0.131	0.157	0.154	0.118	0.126	0.120
	USA	0.066			0.028		
Wheat	China	0.052		0.055	0.032		0.031
	France	0.067		0.072	0.055		0.054
	India	0.061			0.040		
	Russia	0.102		0.109	0.065		0.075
	USA	0.063		0.062	0.048		0.048
Maize	USA from state	0.133	0.133	0.135	0.071	0.070	0.071
Soybeans	USA from state	0.130	0.130	0.133	0.076	0.076	0.079
Wheat	USA from state	0.129	0.129	0.130	0.076	0.076	0.075

Errors in yield data would not bias results but induce uncertainty on the estimated effects, thus leading to a loss of significance as measured by higher p-values exceeding 0.05. For this reason, simulation analyses were performed to check for the influence of measurement error in the FAO yield data on the estimated significance of the highest order term (interaction, quadratic, or linear). For the global models, the highest order interaction terms started to lose significance (p > 0.05 for maize, rice, wheat, and p > 0.1 for soybeans) after adding approximately 15% noise on maize yields, 10% on rice and soybeans yields, and 25% on wheat yields (S2 Fig). After adding 50% of noise, the highest order terms remained still significant in ~ 65% of the models for maize, 40% for rice, 25% for soybeans, and 75% for wheat. For models at the country level, results varied more among countries and crops (S3 Fig). Highest order terms lost significance on average after adding 18% noise, while at 50% noise, ~ 65% of the highest order terms remained significant.

## Discussion

According to the FAO, maize, rice and wheat account for more than two thirds of the world's food energy intake, albeit with varying importance across regions. The top five producing countries account for roughly half of the global yield for maize (57%) and rice (56.2%), whereas soybean production is more concentrated (84.6%) and wheat production more distributed (43.9%). Thus impacts of climate variability on these crops in the top producing countries should have sizeable downstream effects on the global population. The two most populated countries, China and India, are among the top 5 producers of all 4 crops studied, the USA for 3 and Brazil for 2.

### Yield effects of climate variability

The demonstrated assessment of the nonlinear combined effects of temperature and drought (SPEI) on crop yields focused on climate variability. Thus corresponding estimated percentages of changes reported in this study should be regarded as indicators of sensitivity and ultimately vulnerability. This lies in contrast to the interpretation of reported yield changes in recent decades which depend on perceived climate trends (see e.g. [2]). Consequently, whereas previous studies revealed temperature linked changes in crop yields, this study did not find significant global effects of temperature for maize and soybean yields for average SPEI, but significant effects of drought that were further aggravated in the presence of high temperatures. These combined effects significantly decreased yields of maize, soybeans, and wheat by 11.6, 12.4, and 9.2%, respectively. Among the top producers, maize and soybean yields were predominantly affected by drought (reductions of maize yields between 3.0 and 10.4%, soybeans 3.1 to 12.2%), whereas for wheat higher temperatures were more important (yield reductions between 3.5 and 13.5%). Rice was least affected by climate variability, both in terms of significant global and regional effects as well as via effect sizes. A more detailed comparison of the single crop results to previous studies underlines the strong variation in sensitivities, both between crops and regions.

Maize yields in the USA have been reported to respond strongly to drought [27,28], and in addition to extreme temperatures [14]. In contrast, this study did not find such a strong temperature link (only -4.1%), probably because SPEI already incorporates evapotranspiration, thus accounting for higher evaporative demand as well as temperature induced soil water depletion, the main source of the yield decreases associated with extreme heat [14]. The negative effect of too wet conditions found in this study may potentially be due to flooding and heavy precipitation events having caused lack of soil aeration or crop damage [29].

It is reported that China maize yield variability depends on temperature and precipitation variability, although there is large spatial variability [30]. Hence, the use of country averages might have obscured variability effects in this study where dry conditions only amounted to a yield reduction of 3.0%. Increased precipitation has been linked to higher yields of maize in Brazil [31,32]. In this study, above average SPEI was not associated with increased yields, but below average SPEI was associated with a 9.1% yield reduction.

The large and highly significant effect of temperature variability on maize in Ukraine might be linked to its continental climate with large temperature variations [33]. However, the effect found in this study was rather large (-8.6%) compared to other regions in Europe as well as in global studies [11,34]. Based on only 23 years of data, the estimates should be taken with caution.

Maize is grown in many parts of India with diverse climates: in the north the yield is mostly temperature dependent and in the rest it is mostly precipitation and only partly temperature dependent [8]. Therefore, country averages need to be interpreted carefully. Nonetheless, findings from this study (-12.7% for dry and hot conditions) match those of [35], where reductions in precipitation were shown to be harmful to maize yields for high temperatures, while increases in precipitation benefitted maize yields.

For rice, previous global studies similarly encountered the large uncertainties and small effect sizes observed here [2,11]. In northern China, temperature variability was linked to rice yields, while in the central and southern parts precipitation was mainly limiting [8]. Also depending on the region, precipitation was correlated positively or negatively to rice yields [30], which could possibly explain, why in this study of country averages, both dry and wet conditions were associated with increased yields.

Rice is planted widely across India. In rainfed areas rice yields have been linked to precipitation variability, and in irrigated areas, to temperature and partly also to precipitation [8]. Increased temperatures were associated with yield decreases [36], similar to the findings of this study. However, the influence of climatic variables differed strongly for winter and monsoon rice [37], suggesting the need for more detailed analyses on the multiple growing seasons.

For Indonesia, no significant effects of climate variability were found in this study, which may be due to data quality issues. Significant associations between temperature and yield variability have recently been reported for sub-country data [8] and significant negative impacts of temperature have been found in the vicinity of Indonesia, for trial farms in the Philippines, for example [38].

Rice in Bangladesh is heavily irrigated, so temperature has been found to be more important [8], and with positive impacts [39]. However, some areas are still prone to drought [40]. The strong decreases in yield for extreme wet conditions found in this study may be due to extreme events, such as cyclones, that are more frequent in these regions of the world [41,42].

For rice in Viet Nam, negative effects of temperature and positive effects of rainfall have been reported in a regional study [43], with impacts also dependent upon the wet- and dry-season [44]. These findings concur with the large effects of high temperature under wet and dry conditions revealed in this study.

In central and eastern parts of the USA, temperature variability has been found to be the main driver of soybean yield variability, while for other parts of the country, precipitation and precipitation combined with temperature have served as the primary drivers [8,12]. This study did not find a temperature effect on country averages, which may be in part because heat induced soil water depletion might be accounted for by the SPEI. However, based on state-level data there is a temperature effect of -5.9% for warm conditions.

The strong effects of SPEI variability on soybeans in Brazil (-8.1%) found in this study are in concordance with water supply being the main limiting factor [45,46]. Regional studies of soybean yields in Argentina showed that high temperatures and precipitation were the major influence on soybean yields [31,47], which matches the significant effects of both temperature and SPEI found in this study. While soybeans growing in the northern parts of China were mostly drought affected [48], in the southern growing regions they also depended on temperature in addition to precipitation [8,49].

Strong effects of heat and rainfall on soybean yields in India (-11.8% and -12.2%) are in concordance to a regional study [50]. However, to our knowledge, the positive effects from interactions of high temperatures under wet conditions found in this study are new.

Wheat is grown in large parts of China. In central China wheat yields have been associated with precipitation variability [8,30]. However, such effects were non-significant and perhaps not detectable at the country level as used in this study.

Extreme heat is a major factor determining wheat yields in India [51,52], also confirmed by this study, and the lack of a significant link to SPEI in this study could be because wheat is almost completely irrigated (96% as of 2013, [53]). Russia’s wheat producing area is concentrated in the central and eastern part, which is heavily affected by heat and drought [54,55]. No significant link to SPEI was found in this study, which could be due to the country level analysis as well as the low number of years (22) of data.

According to the literature, wheat growing in the USA was found to depend largely on precipitation [8,56], similar to the findings of this study.

Adverse effects of wet conditions on wheat yields in France, as found in this study, seem counterintuitive at first glance, but could be caused, for example, by negative effects of soil moisture at planting and harvesting, or waterlogging during dormancy [57].

When comparing sensitivities among the major crop producers, it is notable that India appeared to be more vulnerable to drought for growing maize, rice and soybeans than China, whereas the temperature sensitivity of wheat was comparable. India and China are both large countries with similar population size and food consumption habits, however China has less cultivated area, uses less fertilizer and has a higher per area productivity. Most likely, the higher proportion of rainfed agriculture in India (for maize ~80% in India versus ~50% in China, for rice ~43% in India versus virtually none in China, see [53]) results in the lower productivity as well as in the higher vulnerability to drought revealed by this study. A point which can not be clarified in this study is whether short-lived pollutants such as ozone contributed to the yield losses in dry and hot summers, since intensive trophospheric ozone formation is most prone to such weather situations. Such toxic substances directly impact crop growth, for example, black carbon and ozone were identified as major factors for rice and wheat yield losses in India [58].While climate intensification effects, that is interactions between current and previous year climate, have been proposed a few years ago [19], we are unaware of any studies incorporating them. This study found mostly links of previous year climate with rice yields, and some for soybeans and wheat. It should be noted that, for example, multiple growing seasons might cause spurious effects of previous year climate, so while this study serves as an initial effort to characterize climate intensification, future studies should account for multiple growing seasons more carefully.

### Sustainability and food security

The green revolution led to large increases in crop yields worldwide since the 1960s [59], due to the adoption of new varieties, fertilizers, pesticides, and increased mechanization. While anthropogenic input and management played, and still play, a key role in sustaining long-term trends in crop yields [60], year-to-year variation of yields is largely determined by weather [9]. And while crop yields increased globally since the 1960s, crop yield variability did not increase, on the contrary, it primarily decreased [9]. Nevertheless, climate variability causes large fluctuations in crop yields, and with climate change a new player enters the stage of determining long-term crop yields.

Staple crops cover large parts of the human diet, and higher variability in yields leads to less stable production, higher price fluctuations and smaller incomes for food producers. The strong vulnerability of global maize, soybeans and wheat yield to combined effects of heat and drought as revealed in this study will threaten food security in the long run under progressing climate change. For most crops, the significant climate impacts affect all top producers. Consequently, only regionally alternating extreme events may level out the worst consequences. However, even then, regional effects on local prices are still likely, a second factor threatening food security.

Achieving food security is the second of the UN sustainable development goals. The global ecological footprint of agriculture stands in the way of sustainably fulfilling the increasing demand for food. Much research has been devoted to closing yield gaps, that is the difference between the actual yields and the potential yields given same climatic constraints [61–63]. This would eliminate the need for agricultural expansion by managing the existing agricultural areas better, for instance by increasing nutrient and water efficiency [61], or by spatially reallocating crops to where they are economically best profitable [62].

The immense need of water for agriculture, combined with massive groundwater depletion [64], and climate change induced water scarcity [65], calls for additional measures, such as improving crop water productivity or crop water use efficiency [66], which would increase yields, and at the same time provide more water for people and ecological services.

Future climatic variability, which implies more heat and drought, could be coped with by breeding and improving crop varieties such that they have an increased tolerance to heat and drought stress [67] and adapting planting dates [68]. But while these are yet theoretical ideas, practical implementations, such as conservation agriculture, could already be used to deal with these issues.

Conservation agriculture, compromising minimal soil disturbance, permanent soil cover and appropriate rotation, can reduce canopy temperature, increase water efficiency, reduce greenhouse gas emissions, and could also be more profitable from an economic perspective, however, it requires high initial investments in new machinery and high levels of skill and knowledge [69].

### Limitations

Some caveats should be noted. Using a mean growing season climate obscures effects of intra-seasonal effects, such as short heat waves or dry periods in critical plant growth stages. Intra-seasonal climate variability was reported to have different impacts depending on the timing of the events [57]. Since intra-seasonal weather has been averaged over in the calculations of this study, the reported estimates might be seen as indication of the climate variability effects for the whole plant growing period. Similarly, the use of country averages obscures regionally varying impacts. However it allows the assessment of the effects of climate variability on crop yields globally. In order to have at the same time a spatially finer resolved assessment, much more detailed data would be required.

Another limitation of this study is that it did not control for other factors affecting yield variability, such as agricultural management practices, pests, socioeconomic conditions, and conflicts. How much water is available to crops can strongly be influenced by irrigation, which can alleviate the impacts of extreme temperatures [16]. Besides that, water availability is related to soil properties and the management thereof [70–72], however, these could be assumed to be mostly independent of climate variability. The occurence of pests on the other hand is related to climate and climate variability [73,74]. The growing of crops and the socio-economy are closely linked and interdependent [75], especially in areas where agriculture is the main source of livelihood. While climate variability may also directly affect the socio-economic conditions, its main effects are on crop yields. The socio-economic conditions, such as supply chain infrastructure, market availability, labour and health issues, then act on top of the effects of climate variability, and can both enhance or reduce the effects of climate variability [76,77].

Since the focus of this study was on year-to-year climate variability and not climate change, long-term trends in both crop yields and climate were removed, such that time is not a confounding variable anymore. Consequently, impacts of climate change on crop yields [78] or impacts of climate change on climate variability [9] could not be considered.

## Conclusion

Using a random effects specification, the multitude of spatial observations on a short time scale were leveraged to determine the interacting climate variability effects on global crop yields from country data, or country yields from sub-country data. In order to estimate detailed interaction effects, sub-country data were necessary to estimate country sensitivities.

Interactions between temperature and SPEI led to a stronger temperature sensitivity of the global maize and wheat yields in dry than normal conditions, and no temperature sensitivity of global soybean yields for wet compared to normal conditions. Using state-level data, USA yields of maize and soybeans were more sensitive to temperature in dry than normal conditions, and soybean yields were less sensitive to temperature in wet than normal conditions. Furthermore, for rice in Viet Nam and soybeans in the USA, consecutive dry years additionally reduced yields, as did consecutive warm years for USA soybeans.

Climate variability accounts for large parts of yield variability, and by not accounting for interactions between temperature and moisture, the effect of temperature on yields might be overestimated in wet conditions and underestimated in dry conditions.

## Supporting information

S1 Fig Climate variability effects on crop yield variability for the USA, determined by national level yield aggregated from state level data. The same as Fig 6, but here effects were estimated from national level yield data as opposed to state level yield data in Fig 6.
(TIFF)

S2 Fig Significance of highest order term in mixed model of global yields after adding random noise on FAO data. Shown on the y-axis is the percentage of significance levels of the highest order interaction term (listed at the top of each panel) for each crop depending on noise level added to yields (on the x-axis). Generally speaking, as one adds more noise to the yields (moves to the right of the x-axis) the interaction term becomes less statistically significant, e.g. higher amounts of red indicating p-value > 0.05.
(TIFF)

S3 Fig Significance of highest order term in linear models of country yields after adding random noise on FAO data. Shown is the percentage of significance levels of the highest order terms (interaction, quadratic, or linear, as listed at the top of each panel) for each crop and country depending on noise level added to yields.
(TIFF)

S1 Table Time series length of FAO yield data: After (before) quality checks. Maximum number 54 corresponds to full time series length (1961–2014). Quality criteria: if countries reported the identical values in 2 consecutive years, then all years prior or after were excluded; manual visual inspection (see methods for further details).
(DOCX)

S2 Table Country crop time series that needed higher dimension for yield detrending. See methods for details.
(DOCX)
