# Supplementary figures and images for: Interactions between temperature and drought in global and regional crop yield variability during 1961-2014
Source: PLoS One. 2017 May 26;12(5):e0178339. doi: 10.1371/journal.pone.0178339 (PMC5446168; doi:10.1371/journal.pone.0178339)

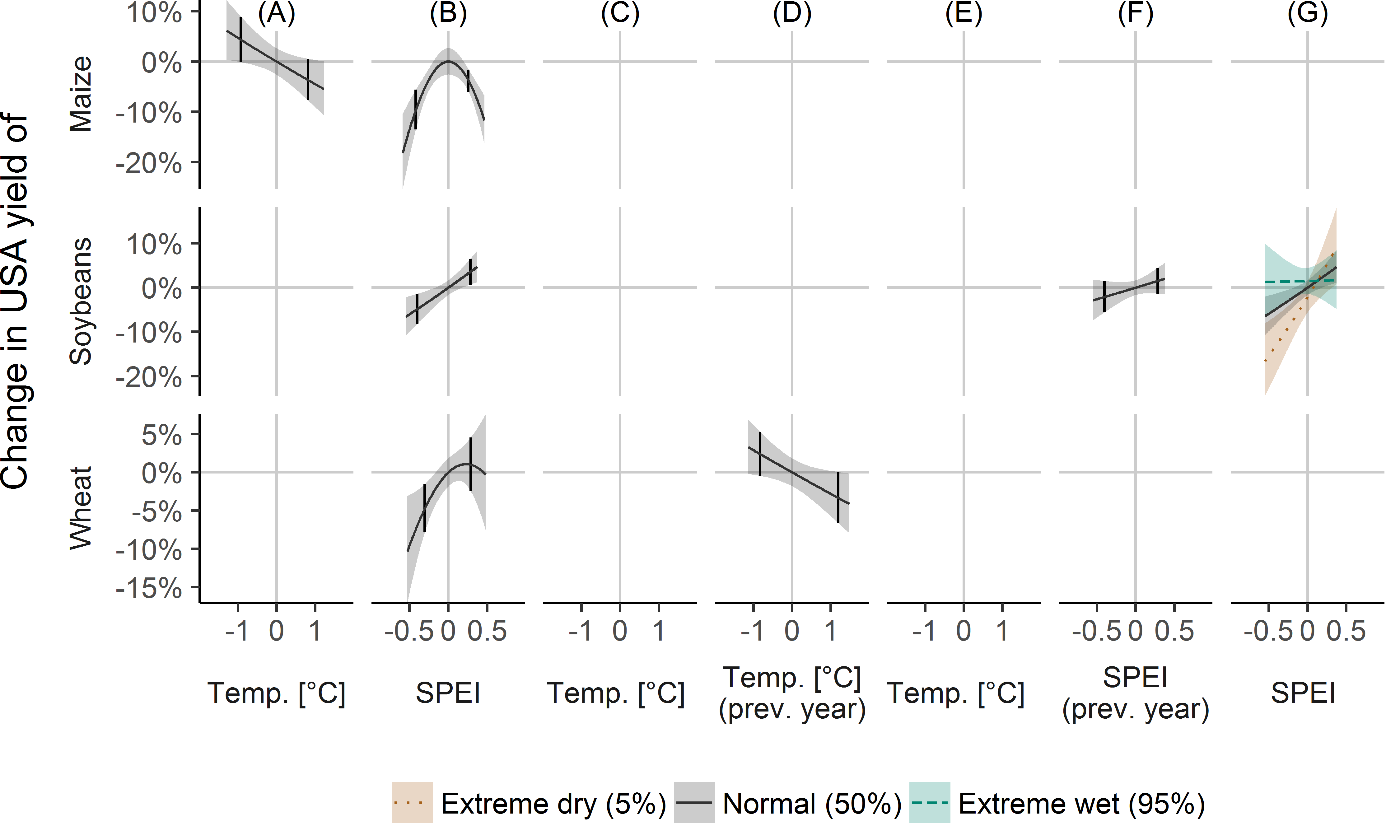

Supplement: S1 Fig — The same as Fig 6, but here effects were estimated from national level yield data as opposed to state level yield data in Fig 6. (TIFF) [file pone.0178339.s001.tiff]

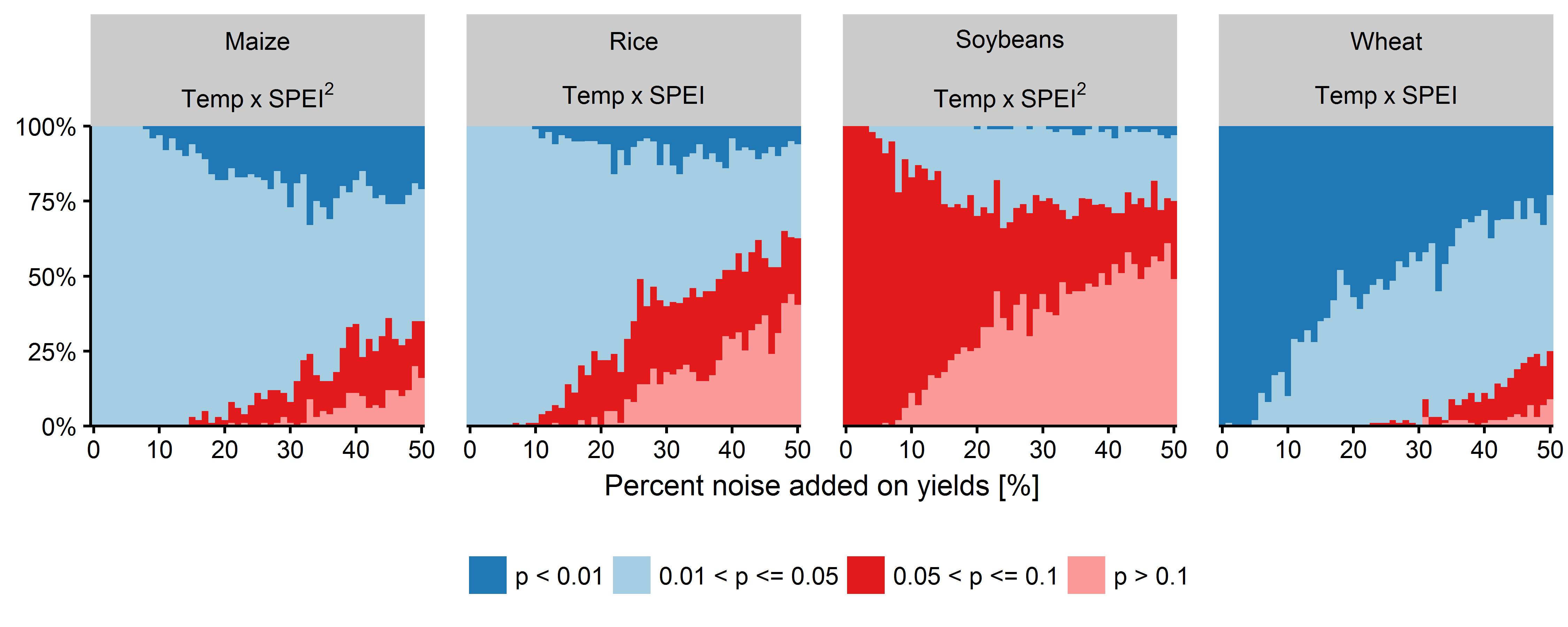

Supplement: S2 Fig — Shown on the y-axis is the percentage of significance levels of the highest order interaction term (listed at the top of each panel) for each crop depending on noise level added to yields (on the x-axis). Generally speaking, as one adds more noise to the yields (moves to the right of the x-axis) the interaction term becomes less statistically significant, e.g. higher amounts of red indicating p-value > 0.05. (TIFF) [file pone.0178339.s002.tiff]

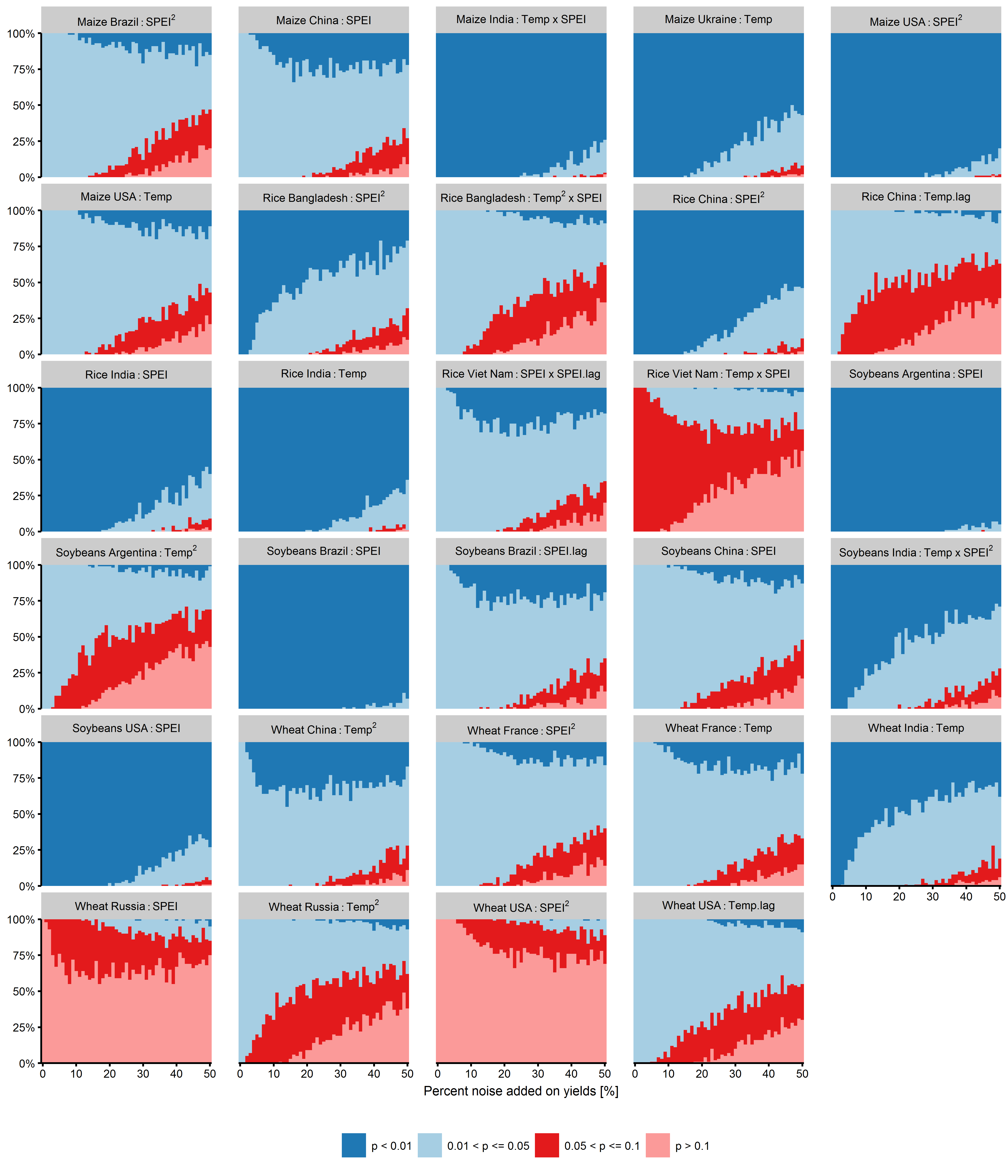

Supplement: S3 Fig — Shown is the percentage of significance levels of the highest order terms (interaction, quadratic, or linear, as listed at the top of each panel) for each crop and country depending on noise level added to yields. (TIFF) [file pone.0178339.s003.tiff]
