# Supplementary material for: Interactions between temperature and drought in global and regional crop yield variability during 1961-2014
Source: PLoS One. 2017 May 26;12(5):e0178339. doi: 10.1371/journal.pone.0178339 (PMC5446168; doi:10.1371/journal.pone.0178339)
Supplement: S1 Table — Maximum number 54 corresponds to full time series length (1961–2014). Quality criteria: if countries reported the identical values in 2 consecutive years, then all years prior or after were excluded; manual visual inspection (see methods for further details). (DOCX) [file pone.0178339.s004.docx]

**S1 Table. Time series length of FAO yield data: after (before) quality checks.** Maximum number 54 corresponds to full time series length (1961-2014). Quality criteria: if countries reported the identical values in 2 consecutive years, then all years prior or after were excluded; manual visual inspection (see methods for further details).

| Country | Maize | Rice | Soybeans | Wheat |
| --- | --- | --- | --- | --- |
| Argentina | 51 (54) | - | 54 (54) | 54 (54) |
| Australia | - | - | - | 54 (54) |
| Bangladesh | - | 54 (54) | - | - |
| Brazil | 54 (54) | 54 (54) | 54 (54) | - |
| Canada | - | - | 54 (54) | 54 (54) |
| China | 54 (54) | 54 (54) | 54 (54) | 54 (54) |
| France | 53 (54) | - | - | 54 (54) |
| Germany | - | - | - | 54 (54) |
| Hungary | 54 (54) | - | - | - |
| India | 54 (54) | 54 (54) | 43 (54) | 54 (54) |
| Indonesia | 54 (54) | 54 (54) | - | - |
| Iran (Islamic Republic of) | - | - | - | - (54) |
| Italy | 54 (54) | - | - | 54 (54) |
| Japan | - | 54 (54) | - | - |
| Kazakhstan | - | - | - | 23 (23) |
| Mexico | 54 (54) | - | - | - |
| Myanmar | - | 54 (54) | - | - |
| Pakistan | - | - | - | 54 (54) |
| Paraguay | - | - | 45 (54) | - |
| Philippines | - | 54 (54) | - | - |
| Poland | - | - | - | 54 (54) |
| Republic of Korea | - | 54 (54) | - | - |
| Romania | 54 (54) | - | - | 54 (54) |
| Russian Federation | - | - | - | 23 (23) |
| South Africa | 54 (54) | - | - | - |
| Thailand | - | 54 (54) | - | - |
| Turkey | - | - | - | - (54) |
| Ukraine | - | - | - | 23 (23) |
| United Kingdom | - | - | - | 24 (54) |
| United States of America | 54 (54) | 54 (54) | 54 (54) | 54 (54) |
| Viet Nam | - | 54 (54) | - | - |
| Total | 644 (648) | 648 (648) | 358 (378) | 741 (879) |
