# Supplementary material for: Interactions between temperature and drought in global and regional crop yield variability during 1961-2014
Source: PLoS One. 2017 May 26;12(5):e0178339. doi: 10.1371/journal.pone.0178339 (PMC5446168; doi:10.1371/journal.pone.0178339)
Supplement: S2 Table — See methods for details. (DOCX) [file pone.0178339.s005.docx]

**S2 Table. Country crop time series that needed higher dimension for yield detrending.** See methods for details.

| Country | Crop(s) |
| --- | --- |
| China | Rice, Soybeans, Wheat |
| Indonesia | Rice |
| Italy | Maize |
| Mexico | Maize |
| Myanmar | Rice |
| Philippines | Rice |
| USA | Rice |
| Viet Nam | Rice |
